# Hearing Loss Screening, Diagnosis, and Treatment for Refugees and Asylees in an Urban Clinic, 2014-2017

**DOI:** 10.1177/2473974X221132509

**Published:** 2022-12-16

**Authors:** Katrin Jaradeh, Elizabeth Liao, Cristy Dieterich, Sammi Truong, Payal Anand, Dylan K. Chan, Eva Raphael

**Affiliations:** 1 School of Medicine, University of California, San Francisco, San Francisco, California, USA; 2 Newcomers Health Program, San Francisco Refugee Health Assessment Program, Community Health Equity and Promotion Branch, San Francisco Department of Public Health, San Francisco, California, USA; 3 Department of Otolaryngology–Head and Neck Surgery, University of California, San Francisco, San Francisco, California, USA; 4 Department of Epidemiology and Biostatistics, University of California San Francisco, San Francisco, California, USA; 5 Department of Family and Community Medicine, University of California San Francisco, San Francisco, California, USA

**Keywords:** refugee, immigrant, hearing loss, audiology, disparities

## Abstract

**Objectives:**

First, to determine whether using a single-question subjective hearing screen vs gold standard audiometric evaluation is effective for hearing loss screening in refugees and asylees. Second, to understand the clinical pathways for hearing loss diagnosis and treatment.

**Study Design:**

This is a case series with chart review from January 2014 to December 2017.

**Setting:**

A large urban safety net primary care clinic in San Francisco, California.

**Methods:**

Patients were included who had a medical record and completed single-question subjective hearing screening and audiometric evaluation during refugee health examinations. An overall 349 patients met all inclusion criteria.

**Results:**

Out of 349 patients, 48% were male; the median age was 29.3 years (SD, 15.1). The majority came from Central or South America (n = 148, 42%) and China (n = 79, 23%). Among all patients, 10 (3%) failed the subjective hearing screen, and 18 (5%) failed audiometric evaluation. Of those who failed the subjective hearing screen, 4 (40%) passed audiometric evaluation. Of those who failed the audiometric evaluation, 12 (66%) passed subjective screening, and only 5 (28%) received a diagnostic audiogram, with 4 diagnosed with hearing loss and 1 receiving hearing aids. The sensitivity of the subjective screening question was 33% and the specificity 99% as compared with audiometric evaluation.

**Conclusion:**

Audiometric evaluation is relatively inexpensive and easily administered, while a single subjective question is a poor screening tool. Hearing loss is undertreated in this population. Ensuring appropriate hearing loss screening, diagnosis, and treatment in this population is paramount to improving quality of life.

Hearing loss (HL) affects 430 million people worldwide, or 5% of the world’s population.^1,2^ Eighty percent of those affected by HL live in low- or middle-income countries.^1^ There are many causes of HL, such as congenital conditions, genetic causes (eg, Pendred syndrome), vaccine-preventable infections, age-related degenerative processes, and head injury.^3,4^ HL has been shown to have significant negative impacts on quality of life, communication, and psychosocial health. It is the second-highest contributor to years living with disability, after depression.^2,5-8^

Forcibly displaced populations, who are often fleeing conflict zones, account for 1% of the world’s population. At the end of 2020, this included 84 million people.^9^ The prevalence and incidence of HL in this population are not well studied, and the majority of studies have focused on newborn hearing screening.^10,11^ Few have investigated HL in postnatal pediatric and adult populations, despite a recent study showing that the prevalence of sensorineural HL is significantly higher in Syrian refugee children than Turkish natives.^12^ In addition, this population has a higher prevalence of preventable and treatable infections and lack access to high-quality medical care, which places them at a higher risk for HL.^13-15^

Undiagnosed and untreated HL can have a severe impact on quality of life. Depression, anxiety, and low self-esteem have been associated with severe HL, especially among young men and women.^16,17^ Refugees and asylees are exposed to various physical and emotional stressors before, during, and after their migration, which affects their mental health.^18^ The high prevalence of mental health conditions among patients with HL may be compounded in the refugee and asylee population, which has a higher prevalence of mental health conditions when compared with host populations.^18^

Between 2013 and 2017, >438,000 refugees were admitted and >121,000 people were granted asylum in the United States.^13^ All refugees and asylees who arrive in the United States are eligible to receive a refugee health examination within 90 days of arrival.^19,20^ Guidelines from the Centers for Disease Control and Prevention for this examination include screening for communicable and vaccine-preventable diseases, as well as assessing for hearing and vision.^21^ Hearing screening can be performed in 2 ways. Single-question subjective hearing screen involves asking individuals to report if they have difficulty hearing. It has significant limitations, such as low sensitivity for detecting mild HL, as well as language and cultural barriers.^11,22^ The second method is through an audiometric evaluation, a validated objective measure to determine if a person can hear specific calibrated tones. Audiometric evaluation is considered the gold standard when screening for HL.^22,23^ However, institutions adapt and develop their own screening protocols, including hearing screens, which are not often standardized.^22^

In this study, we assessed differences between subjective hearing screen and gold standard audiometric evaluation in detecting HL in the refugee and asylee population. We used data from 2 sources: refugee health examination data from the Office of Refugee Health’s Refugee Health Electronic Information System (RHEIS; California Department of Public Health) and electronic health record (eHR) data from a large urban safety net primary care clinic. We also describe the clinical pathway for diagnosing, evaluating, and treating HL in this vulnerable population.

## Methods

### Study Design and Setting

This is a case series with chart review of health examinations for refugees, asylees, and victims of trafficking that were conducted at a large urban safety net primary care clinic in San Francisco between January 2014 and December 2017. Data were analyzed from October 2021 to January 2022. This study was approved by the Institutional Review Board of the University of California, San Francisco (17-22184).

The refugees and asylees who arrive in San Francisco County are enrolled in the San Francisco Department of Public Health’s Newcomers Health Program (NHP), nested within the primary care clinic. The NHP is staffed by trained health workers and interpreters who culturally and linguistically reflect the populations served. There, health workers provide health screens and health coaching for forcibly displaced individuals and connect them to community resources.^24^

### Patients

**Figure 1.**
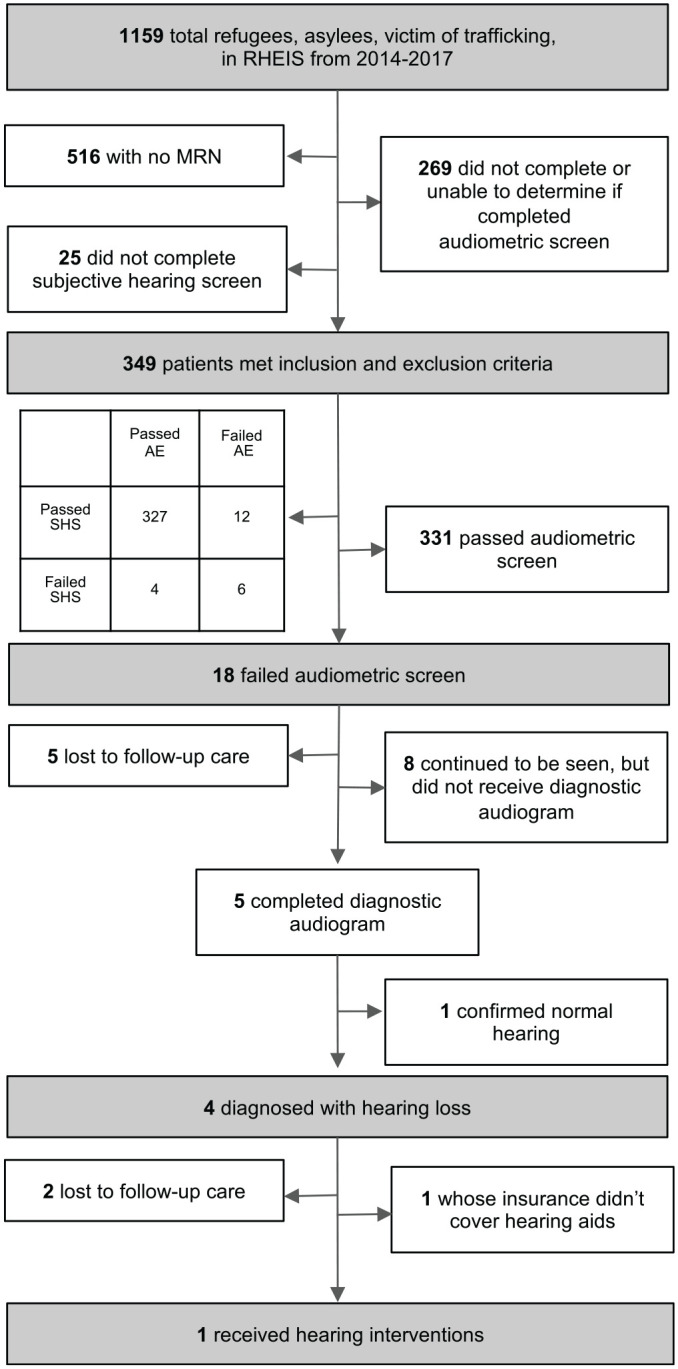
Inclusion of subjects, hearing loss screening, diagnosis, and treatment. Patients charts from 2014 to 2017 in RHEIS were reviewed. Results of hearing screen, audiogram, and treatment were included. AE, audiometric evaluation; MRN, medical record number; RHEIS, Refugee Health Electronic Information System; SHS, subjective hearing screen.

There were 1159 patients in California’s RHEIS from 2014 to 2017. Patients met inclusion criteria if they had a medical record number and completed a subjective hearing screen and if their refugee health examination records included results from their audiometric evaluation (**Figure 1**). We defined refugees and asylees as people who leave their home as a result of persecution, conflict, violence, or human rights violations^10,11^; refugees apply for their status in their home country, while asylees apply in the host country.^10,11^ We define victims of trafficking as people who are recruited and forced or coerced into commercial sex acts or labor.^12^ Here, we report on refugees, asylees, and victims of trafficking as refugees and asylees.

### Data Sources and Measurement

The refugee health examination is conducted over 2 visits. During the first visit, NHP staff collect sociodemographic and medical history as well as current symptoms, including subjective hearing screen, through a standardized culturally and linguistically appropriate form created by the California Department of Public Health’s Office of Refugee Health, following guidelines from the Centers for Disease Control and Prevention. Data from this form are entered into RHEIS, a web-based database developed in 2013 to transmit, standardize, and generate reports on refugee health screening data.^25^ Clinicians conduct a physical evaluation, and patients are evaluated for communicable and chronic diseases, as well as mental health conditions: depression, anxiety, and posttraumatic stress disorder. During the second visit, clinicians review and discuss laboratory results with the patient, and referrals are made when needed.

Subjective hearing screen and audiometric evaluation are performed by NHP staff in the patient’s native language, with an in-person or phone interpreter if needed. Patients are asked, “Do you have difficulty hearing?” to evaluate for subjective HL, where a patient answering “yes” indicates a failed screen. Patients then undergo an audiometric evaluation in a private examination room with minimal background noise. The audiometric evaluation is conducted at 25 dB with a calibrated audiometric machine (model MA27; MAICO) at the following frequencies: 500, 1000, 2000, and 4000 Hz. Each ear is tested separately, and the examination lasts 2 to 5 minutes. Failing audiometric evaluation is defined by not responding to 25 dB at ≥1 frequencies in either ear.

Patients who fail audiometric evaluation are referred to the audiology department within the same public health care system if they live in San Francisco. Those who reside in another county are recommended to follow up with their primary care providers. Patients seen in the audiology department receive a diagnostic audiogram by a trained audiologist. If the patient is diagnosed with HL, a referral is made to an otolaryngologist.

### Outcome Variables

Demographic characteristics, medical history, and subjective hearing screen data were extracted from RHEIS. Audiometric evaluation results, diagnostic audiograms, and follow-up data (ie, referral to an otolaryngologist) were collected manually through chart abstraction from the eHR. Patients who failed audiometric evaluation were categorized into 3 groups: (1) received a diagnostic audiogram, (2) continued to be seen but did not receive diagnostic audiogram, and (3) were lost to follow-up. Loss to follow-up was defined as <1 visit to the clinic within 1 year following the refugee health examinations.

### Statistical Methods

We calculated the prevalence of HL based on the number of patients diagnosed within the cohort who met the inclusion criteria. Descriptive statistics, including frequencies and percentages, were used to summarize categorical variables, and median and standard deviations were used for continuous variables.

The differences between patients who failed or passed subjective hearing screen and/or audiometric evaluation were compared with the Fisher exact test.

## Results

### Patient Demographic Characteristics

Between 2014 and 2017, 1159 refugees and asylees received health screens through the NHP. Of the 1159 patients, 349 people met inclusion criteria. Of these, 47.9% were male, and the median age was 29.3 years (SD, 15.1 years). The majority of patients reported preferring a language other than English, with 171 (49.0%) preferring Spanish, 22 (6.3%) Arabic, and 78 (22.3%) Mandarin or Cantonese. Many patients (n = 148, 42.4%) were from Central or South America, 79 (22.6%) were from China, and 48 (13.8%) were from the Middle East and North Africa or sub-Saharan Africa. Posttraumatic stress disorder (n = 27, 7.7%), depression (n = 24, 6.8%), and anxiety (n = 11, 3.2%) were the top 3 mental health diagnoses in this cohort (**Table 1**).

**Table 1.** Demographic Characteristics of All Refugees and Asylees in a Large Urban Safety Net Clinic and the Study Cohort of Refugees and Asylees.^a^

	All refugees and asylees (n = 1159)	Study cohort (n = 349)
Sex: male	590 (50.9)	167 (47.9)
Preferred language		
English	18 (1.5)	3 (0.9)
Spanish	400 (34.5)	171 (49.0)
Mandarin and Cantonese	218 (18.8)	78 (22.3)
Arabic	114 (9.8)	22 (6.3)
ASL	1 (0.1)	0 (0.0)
Other	331 (28.6)	75 (21.5)
Unknown	77 (6.6)	0 (0.0)
Age, y, median ± SD	33.1 ± 15.4	29.3 ± 15.1
0-20	236 (20.4)	80 (22.9)
21-40	555 (47.9)	172 (49.2)
41-60	302 (26.1)	81 (23.2)
>61	59 (5.1)	16 (4.6)
Unknown	7 (0.6)	0 (0.0)
Comorbidities		
Diabetes	15 (1.3)	5 (1.4)
HTN	25 (2.2)	8 (2.3)
PTSD	59 (5.0)	27 (7.7)
Depression	52 (4.5)	24 (6.8)
Anxiety	24 (2.1)	11 (3.2)
Region of origin		
North America	0 (0.0)	0 (0.0)
Central or South America	368 (31.7)	148 (42.4)
Mexico	70 (6.0)	25 (7.2)
Asia or South Pacific	190 (16.4)	41 (11.7)
China	220 (18.9)	79 (22.6)
MENA or sub-Saharan Africa	221 (19.0)	48 (13.8)
Europe	61 (5.3)	8 (2.3)
Unknown	29 (2.5)	0 (0.0)

Abbreviations: ASL, American Sign Language; HTN, hypertension; MENA, Middle East and North Africa; PTSD, posttraumatic stress disorder.

a Values are presented as No. (%).

### Subjective and Audiometric Hearing Screen Results

Of the 349 patients, 10 (2.8%) failed subjective hearing screen, and 18 (5.2%) failed audiometric evaluation. Of those who failed the subjective hearing screen, 4 (40%) passed audiometric evaluation. Of 339 people who passed the subjective hearing screen, 12 (3.5%) failed audiometric evaluation (**Table 2**).

**Table 2.** Hearing Loss Clinical Pathway for Patients Who Failed Audiometric Screen.

					Diagnostic audiogram					
No.	Age range, y	Sex	Region of origin	Answer to subjective hearing screen^a^	Completed	PTA: left ear, dB	PTA: right ear, dB	Hearing intervention	Had 2 visits within 6 mo	Details
1	41-60	M	Central America	Y	N			N	N	Experienced homelessness
2	41-60	F	Central America	N	N			N	N	
3	21-40	F	Central America	N	N			N	Y	
4	41-60	F	Central America	N	N			N	N	Non-SF Resident
5	60-69	F	Asia	N	N			N	Y	
6	60-69	F	MENA	Y	N			N	N	Non-SF resident
7	0-20	M	Asia	N	N			N	N	Had 1 visit
8	21-40	M	MENA	N	N			N	N	
9	41-60	M	Asia	N	N			N	Y	
10	21-40	M	Sub-Saharan Africa	N	N			N	N	
11	41-60	F	Asia	N	N			N	N	
12	41-60	F	Asia	N	N			N	N	Declined referral
13	41-60	F	Asia	N	N			N	Y	Declined referral
14	41-60	F	Asia	N	Y	10	10	N	Y	Patient started streptomycin for TB and was monitored for ototoxicity
15	21-40	F	Central America	Y	Y	20	108.75	N	N	
16	21-40	M	MENA	Y	Y	106.25	5	N	Y	Insurance did not cover hearing aids
17	41-60	M	Asia	Y	Y	75	81.25	N	Y^b^	
18	0-20	M	Central America	Y	Y	11.25	16.75	Y		Sensorineural hearing loss, high frequency

Abbreviations: F, female; M, male; MENA, Middle East and North Africa; N, no; PTA, pure tone average; SF, San Francisco; TB, tuberculosis; Y, yes.

a Audiometric screen result: fail for each patient (n = 18).

b No, if counted after diagnostic audio.

The sensitivity of the subjective hearing screen was 33.3% and specificity 98.8% as compared with audiometric evaluation. The positive predictive value for the subjective hearing screen was 60.0% and the negative predictive value 93.7% as compared with audiometric evaluation.

### Clinical Pathway for Evaluation of HL

The clinical pathway for HL diagnosis and treatment was assessed for the 18 patients with a failed audiometric evaluation. Of these 18 patients, 2 (11.1%) had a mental health diagnosis. Only 5 (27.7%) received a diagnostic audiogram, 4 (22.2%) continued to be seen in the primary care clinic but did not receive a diagnostic audiogram, and 9 (50%) were lost to follow-up. (**Figure 1**). Of the 5 patients who received a diagnostic audiogram, 4 (80%) were formally diagnosed with HL. Thus, the prevalence of HL was 1.1% in our cohort of 349 refugees and asylees. The mean pure tone average based on diagnostic audiometry was 27.8 dB for the better-hearing ear and 78.25 dB for the worse-hearing ear (**Table 2**). Of the 4 patients with HL, 2 (50%) were lost to follow-up, and 1 (25%) patient’s insurance did not cover hearing aids. Just 1 (25%) patient subsequently received hearing amplification interventions. The reasons why other patients were not further evaluated included declining a referral or not living in San Francisco (5/18, 27.8%) (**Table 3**).

**Table 3.** Results From Subjective Hearing Screen and Audiometric Screen Among the Study Cohort.^a^

	Audiometric evaluation, No. (%)		
Response to subjective hearing screen	Fail	Pass	Total
Yes	6	4	10 (2.8)
No	12	327	339 (97.2)
Total	18 (5.2)	331 (94.8)	349

a Subjective hearing screen is when a patient is asked, “Do you have difficulty hearing?” to evaluate for subjective hearing loss, and the answer options were “yes” or “no.” A “fail” on audiometric screening was when a patient could not identify a 25-dB sound at 500, 1000, 2000 or 4000 Hz.

## Discussion

This is one of the only reports on hearing screening in refugees and asylees. We found that the commonly used single-question subjective hearing screen is an inadequate screening tool for HL, with a sensitivity of just 33.3%. There is a steep drop-off in the pathway to care for HL in this population, with the majority of patients who screened positive for HL not receiving diagnostic testing. Furthermore, the majority of patients who were diagnosed with HL did not receive hearing amplification. Refugees and asylees have extensive histories of physical and psychological trauma, which can compound the negative impacts of HL on quality of life and lead to worse health outcomes.

### Sensitivity of Hearing Screening

With a sensitivity of only 33.3%, subjective hearing screen is a substandard screening tool for HL in the refugee and asylee population.^26^ In comparison, the sensitivity of the single-question screening for HL in older adults in the United States is between 66% and 80%, close to that of audiometric evaluation with a sensitivity that ranges from 86% to 100%.^10^ Single-question screening for HL has significant limitations in immigrant populations. Language barriers and cultural or other stigma associated with HL may all influence how patients answer, in addition to mild long-standing HL that may not be perceived by the patient. Validated questionnaires have previously been used, such as the Shortened Hearing Handicap Inventory for Elderly, which is used to quantify the emotional and social effects of self-perceived HL.^27^ Unfortunately, this too has severe limitations. For one, validated questionnaires in languages other than English have yet to be created.^28^ For another, there are variations in test thresholds in scoring questions, and for that reason some investigators have concluded that single-question screening is preferable to questionnaires.^29^

Rather than developing new subjective hearing screen tools, audiometric evaluation is an existing tool that meets all the criteria for an acceptable screening test. In addition, it has been shown to be a good approximation of diagnostic audiograms.^11,30^ Possible perceived barriers for audiometric evaluation are the cost of purchasing and maintaining an audiometric machine. However, audiometric machines are widely available. The average cost of a new audiometric machine varies between several hundred to thousands of dollars; similar models to our MAICO model MA27 were priced at <$100 at the time of publication.^31^ Clinics that are affiliated with a hospital can have their machines regularly serviced by their corresponding biomedical engineering departments. Additionally, ambient noise was shown to affect pure tone screening failure rates, thereby placing importance on screening location and headphones.^32^ Current developments in audiometric evaluation include technological advancements, such as Kuduwave,^33^ a South African company that has produced an audiometric machine that allows for screening and diagnostic testing and has been tested in international communities. Future technological advances will continue to make audiometric evaluation more accessible and feasible for a variety of clinical settings.

Our results indicated that many of the patients who were identified with HL on audiometric evaluation did not subjectively report hearing difficulty (12/18, 66%). Indeed, another study showed that 13% of patients did not return for follow-up because they did not think that their HL was serious enough.^34^ HL in many communities is associated with stigma.^35,36^ Furthermore, factors such as age, sex, income, education level, and support systems have been shown to influence one’s perception of subjective HL.^11,37^ A study found that older age was associated with underestimation of subjective HL across sex, race, and ethnicity.^11^ Additionally, one study reported the prevalence of moderate to severe depression among patients with HL to be 11.4%, as opposed to 5.9% for those without HL.^16^ Given the increased prevalence of depression, anxiety, and posttraumatic stress disorder among the refugee and asylee population, timely identification and intervention of HL can have a major impact on quality of life.^2,18,38^ Screening and education about HL is essential to overcoming these barriers.

### Screening Follow-up and Outcomes

The low prevalence of HL in our study is likely an underestimate, most likely due to loss to follow-up at all stages of the clinical pathway. Almost three-quarters of those who were found to have HL on audiometric evaluation did not have a formal diagnostic audiogram. Only 1 patient obtained an amplification device; however, the other 3 patients’ formal audiograms showed severe to profound HL in the worse ear, which may warrant amplification use.^39^ This loss to follow-up may be due to difficulty in accessing care or receiving care out of network, which would not be captured in the eHR, as more than a quarter of the refugees and asylees in our study lived outside of San Francisco county. In addition, 2 patients rejected the referral for further audiometric diagnostic testing. This could be a result of social stigma or lack of perceived benefit. Few studies have assessed reasons for loss to follow-up in immigrant and refugee populations, specifically relating to hearing health. One study found that financial difficulties and needing to be relocated contributed to loss to follow-up for asylum seekers with latent tuberculosis.^40,41^ In a study assessing nonadherence of hearing aids among Hispanic adults with HL, self-reported reasons included thinking that HL was not severe enough, lack of perceived benefits, and concern about affordability.^42^ Our study suggests that there are significant barriers resulting in patients not receiving the appropriate evaluation and treatment for HL.

### Limitations

This study has several strengths. We report HL screening in a large population of refugees and asylees in the largest safety net clinic in a metropolitan city. Clinical services are integrated in this health care system, allowing for assessment of referral to otolaryngologic and audiologic services. There are also several limitations. First, many patients were excluded because of incomplete documentation, either at the examination stage or for audiometric testing. This is in part due to the fact that the health care system in our study transitioned to a new eHR in August 2019, and audiometry records were manually scanned. Second, due to loss of follow-up, we were unable to calculate sensitivity and specificity for audiometric evaluation as compared with diagnostic audiogram results. However, our study cohort was demographically comparable to the entire cohort of 1159 refugees, asylees, and victims of trafficking. Third, this study may have limited generalizability. Refugees who resettled in San Francisco between 2014 and 2017 made up a small proportion (3.7%) of all refugees entering California.^43^ This may limit applicability to the larger refugee/asylee population and other forcibly displaced communities. Nevertheless, drivers of HL and challenges in diagnosing and treating HL may be similar in our study populations seeking care in the United States.

## Conclusion

In a study of HL screening, diagnosis, and treatment in refugees and asylees receiving care in a large urban public primary care clinic, we found that screening for HL using a single-question subjective hearing screen alone is inadequate. A more robust clinical pathway and screening for this population are necessary, including the use of screening audiometric evaluations. Refugees and asylees experience various traumas spanning their migration to a safe country; thus, it is imperative that HL be addressed effectively within this population.

## Author Contributions

**Katrin Jaradeh**, design, conduct, analysis, writing original draft, review, and editing; **Elizabeth Liao**, design, conduct, analysis, writing original draft, review, and editing; **Cristy Dieterich**, conduct, review, and editing; **Sammi Truong**, conduct, review, and editing; **Payal Anand**, conduct, review, and editing; **Dylan K. Chan**, design, conduct, review, and editing; **Eva Raphael**, design, conduct, review, and editing.

## Disclosures

**Competing interests:** None.

**Sponsorships:** None.

**Funding source:** None.
